# Accurate Low Complex Modulation Format and Symbol Rate Identification for Autonomous Lightpath Operation

**DOI:** 10.3390/s22239251

**Published:** 2022-11-28

**Authors:** Diogo Sequeira, Marc Ruiz, Nelson Costa, Antonio Napoli, João Pedro, Luis Velasco

**Affiliations:** 1 Optical Communications Group (GCO), Universitat Politècnica de Catalunya (UPC), 08034 Barcelona, Spain; 2 Infinera Unipessoal Lda., 2790-078 Carnaxide, Portugal; 3 Infinera, 81541 Munich, Germany; 4 Instituto de Telecomunicações, Instituto Superior Técnico, 1049-001 Lisbon, Portugal

**Keywords:** autonomous networking, optical network operation

## Abstract

Network automation promises to reduce costs while guaranteeing the required performance; this is paramount when dealing with the forecasted highly dynamic traffic that will be generated by new 5G/6G applications. In optical networks, autonomous lightpath operation entails that the optical receiver can identify the configuration of a received optical signal without necessarily being configured from the network controller. This provides relief for the network controller from real-time operation, and it can simplify the operation in multi-domain scenarios, where an optical connection spans across more than one domain. Consequently, in this work, we propose a blind and low complex modulation format (MF) and symbol rate (SR) identification algorithm. The algorithm is based on studying the effects of decoding an optical signal with different MFs and SRs. Extensive MATLAB-based simulations have been carried out which consider a coherent wavelength division multiplexed system based on 32 and 64 quadrature amplitude modulated signals at up to 96 GBd, thus enabling bit rates of up to 800 Gb/s/channel. The results show remarkable identification accuracy in the presence of linear and nonlinear noise for a wide range of feasible configurations.

## 1. Introduction

Digital coherent optical transmission, enabled by programmable transponders (TRx) alongside advanced digital signal processing (DSP) algorithms, is nowadays prevalent in optical networks [1,2]; it not only increases spectral efficiency and flexibility, it also simplifies dynamic network operation [3]. Advanced DSP algorithms improve the overall system performance, e.g., by nonlinear interference (NLI) noise equalization [4], and they can extract useful information from the received signals [5]. Furthermore, coherent sliceable-bandwidth variable transponders (S-BVT) enable the configuration of optical signals with a different modulation format (MF) and symbol rate (SR) to deal with different traffic demand scenarios. It is worth noting that, by optimizing the MF and SR, a high energy efficiency can be achieved, as reported in [6].

In the simplest scenario, at the optical connection set-up time, the centralized software defining network (SDN) controller finds the route, the spectrum allocation, and the TRx by selecting the best configuration within the transmitter (Tx) and receiver (Rx) [7]. A centralized monitoring and data analytics (MDA) system [8] can also be responsible for collecting monitoring data from the different nodes analyzing the data and can work together with the SDN controller to perform the required tuning of parameters on the devices [9,10]. Such architecture is further evolving with the development of the Intent-based Networking (IBN) concept [11]. Nonetheless, due to the low latency required, e.g., in 5G and beyond services, optical connectivity of the access to datacenters located in metro/core segments is needed; such connectivity imposes high dynamicity to provide the required capacity and entails coordination amongst the SDN controllers of the different network segments involved. To relieve the SDN controller and other centralized elements from real-time operation, BVTs can make local decisions autonomously, e.g., change the signal’s configuration in terms of the MF and SR to adapt to the actual needs [12,13]. Although these solutions are highly energy efficient, they require a more complex Rx capable of identifying the configuration of the received optical signal.

### 1.1. Related Work

In the literature, several works have proposed different approaches for the MF identification and some of them also include the identification of the SR (e.g., [14]). In particular, most of the previous works use machine learning (ML)-based techniques [15]; these techniques are either supervised, which entails a training phase, or unsupervised, e.g., using clustering algorithms [16]. The authors in [17] presented a deep neural network (DNN) for MF and SR identification considering up to 16 quadrature amplitude modulation (QAM) and slower SRs. In [18], the authors proposed a method based on a random forest for MF identification but also focused on slower SRs, e.g., of up to 16 GBd. As the application of DNNs might increase the complexity of the Rx, the authors in [19] proposed a blind MF identification method based on phase distribution and average amplitude ratio analysis. In addition, the authors in [20] proposed an MF identification algorithm based on clustering for direct detection systems. Graph-theory can be also used for low complex MF identification [21]. Finally, MF identifications based on the Stokes space [22,23,24] are especially attractive because they can be performed before polarization demultiplexing at the Rx DSP block.

Even though some approaches achieve 100% accuracy in the identification of the MF and SR, they do not show that such a level of accuracy can be obtained in higher nonlinear optical transmission scenarios, e.g., in the presence of NLI noise on systems working with high-order MFs and high-speed SRs.

### 1.2. Contributions

In this work, we propose a novel algorithm that can recognize the configuration of the received optical signal, in terms of the MF and SR, by analyzing the received in-phase and quadrature (IQ) optical constellation using the MF and SR supported by the optical transceiver. Its low complexity and simple operation make it ideal for real-time applications and it could be implemented directly in the in-operation TRx. Because high-order MFs and high-speed SRs are more affected by physical layer impairments, such as NLI noise, the proposed algorithm has been tested under realistic scenarios by considering signal configurations of up to 64QAM and 96 GBd, as well as standard single mode fiber (SSMF) and large effective area fiber (LEAF) types.

The remainder of the paper is organized as follows: Section 2 introduces the observed behavior in IQ optical constellations when decoding an optical signal with a different SR and the rationale behind the proposed identification method with an example. Section 3 investigates the effects of decoding the received signal with different SRs. An algorithm that exploits the main observation is proposed to identify the configuration of the received signal. The results of the algorithm evaluation carried out by MATLAB-based simulation are presented in Section 4 for different signal configurations and optical connection scenarios. Finally, Section 5 draws the main conclusions.

## 2. MF and SR Identification Based on the Analysis of the Received IQ Constellation

**Figure 1:**
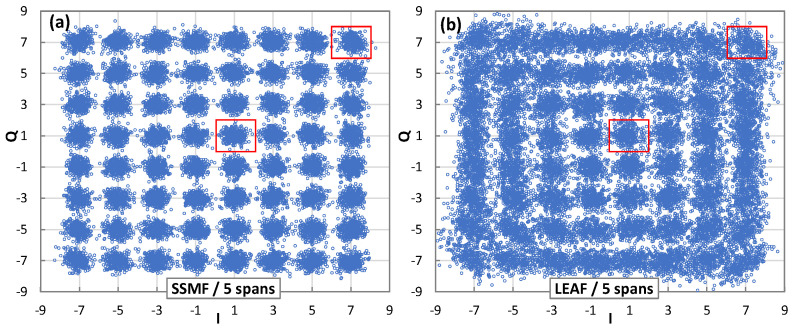
Received 64QAM constellation after 5 SSMF (**a**) and LEAF (**b**) spans.

The proposed method for MF and SR identification relies on the analysis of the received IQ optical constellation when it is decoded with different SRs. In this section, we analyze the effect of using incorrect SRs, which will be useful for the proposed approach. Firstly, we analyze the characteristics of an optical constellation when it is received after optical transmission, i.e., with mux/demux filters, optical amplifiers, and fiber spans. Figure 1 shows several examples of the constellations of a 64QAM/64GBd signal received after a different number of spans; the SSMF spans are presented in Figure 1a and the LEAF spans are presented in Figure 1b. The effect of NLI noise (specifically, self-phase modulation (SPM) and cross-phase modulation (XPM)) can be clearly observed, since the received symbols are not only more dispersed around the *expected constellation point* due to the linear noise, i.e., the amplified spontaneous emission (ASE) noise, but their typical round shape also turns into an elliptical shape. This can be clearly noticed in the outer constellation points at higher-power symbols, e.g., 7 + 7*i*, as opposed to the inner ones, e.g., 1 + 1*i*. Such NLI noise effects make the identification of the MF more challenging.

**Figure 2:**
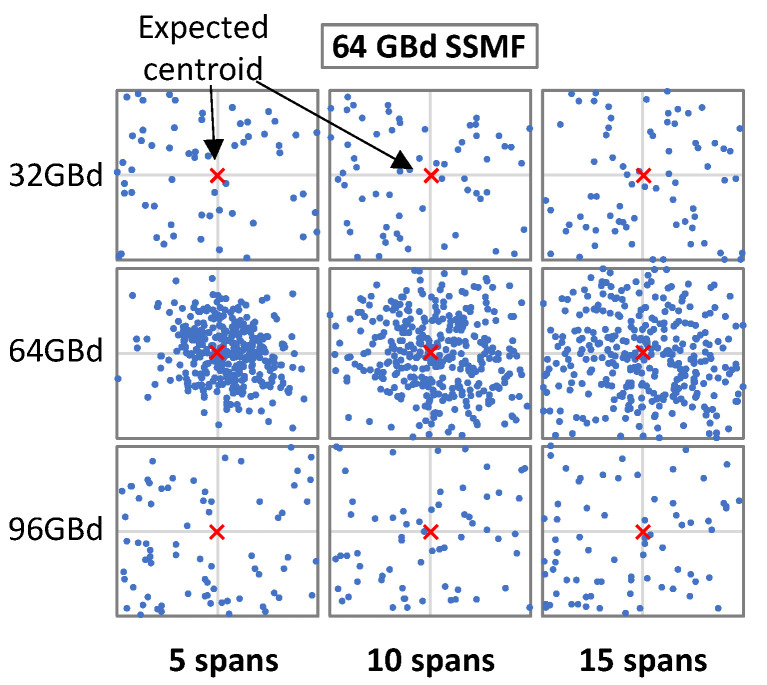
Example of symbols for constellation point 7 + 7*i* decoded with several SRs and for several distances.

However, before the identification of the MF can be carried out, one must estimate the SR. Figure 2 illustrates the effects of decoding signals with different SRs; for the sake of clarity, only the decoded symbols for the constellation point (7 + 7*i*) are shown. Each row in Figure 2 shows the symbols when they are decoded with a particular SR ∈ {32, 64, 96} GBd, whereas the columns show the effect of distance in terms of the number of SSMF spans. We observe that the symbols were more dispersed when the SR used for decoding the signal at the Rx (*SR_Rx_*) was different from the one used for generating the signal at the Tx side (*SR_Tx_*). 

Such dispersion can be quantified by computing the Euclidean distance of every received symbol with respect to the expected constellation point (we refer to it as a *centroid* because of its similarity to clustering algorithms). The average Euclidean distance ${\bar{d}}_{2}\left(S_{SR},C_{MF}\right)$ of a set of symbols *S* decoded with a given SR, with respect to the set of expected centroids *C* for a given MF, can be computed as follows:

(1) $${\bar{d}}_{2}\left(S_{SR},C_{MF}\right)=\frac{1}{\left|S_{SR}\right|}\underset{s\in S_{SR}}{\sum }\underset{c\in C_{MF}}{\min}\left({\left||s-c|\right|}_{2}\right)$$

The average Euclidean distance can be used to determine the correct SR at the Rx side without previous knowledge as will be detailed in the next section.

## 3. Decoding Signals with Different Symbol Rate

In this section, we analyze the effects of decoding an incoming optical signal at the Rx with an *SR_Rx_* different from the one used at the Tx. For the sake of clarity in the discussion, let us assume a back-to-back (B2B) scenario, i.e., without any noise impairments.

The first effect is related to the matched digital filter applied at the optical Rx. In particular, state-of-the-art coherent optical communications systems use root-raised cosine (RRC) filters for Nyquist-WDM spectral shaping [25]; by using an RRC filter at the Tx and the matched RRC filter at the Rx, inter-symbolic interference (ISI) is significantly reduced, and the OSNR is maximized. The frequency response of the RRC filter is $H_{RRC}\left(f|\beta ,T_{s}\right)$, where β is the roll-off factor and *T_s_* = *SR*^−1^ is the symbol period [26]. Thus, assuming ideal matched filters and *SR_RX_* = *SR_TX_*, $H_{RRC_{Tx}}\left(f|\beta ,T_{s}\right)=H_{RRC_{Rx}}(f|\beta ,T_{s})$, and the power spectral density (PSD) of the output optical signal, $S_{out}\left(f\right)$, is

(2) $$S_{out}\left(f\right)=S_{in}\left(f\right)\cdot H_{RRC}{\left(f|\beta ,T_{s}\right)}^{2}=S_{in}\left(f\right)\cdot H_{RC}(f|\beta ,T_{s}),$$

where $S_{in}\left(f\right)$ is the PSD of the input optical signal and $H_{RC}\left(f\right)$ is the frequency response of the raised-cosine (RC) filter.

However, when *SR_Rx_* ≠ *SR_Tx_*,

(3) $$S_{out}\left(f\right)=S_{in}\left(f\right)\cdot H_{RRC}\left(f|\beta ,T_{sTx}\right)\cdot H_{RRC}\left(f|\beta ,T_{sRx}\right)\approx \;S_{in}\left(f\right)\cdot H_{RC}\left(f|\beta ,T_{max}\right)$$

(4) $$T_{max}=max\left(T_{sTx},\;T_{sRx}\right)$$

which introduces a high ISI, especially when *T_sRx_* > *T_sTx_*.

The second effect is related to the sampling step. When *SR_Rx_* ≠ *SR_Tx_*, a different symbol period is used at the Rx (i.e., *T_sRx_* ≠ *T_sTx_*), which produces misalignments in time, and thus leads to higher average Euclidean distances from the received symbols to the expected ones. Special cases are when *SR_Rx_* is a divisor of *SR_Tx_*, i.e., *SR_Rx_* = 1/*a* × *SR_Tx_*, *a* ∈ ℤ ^+^, and, e.g., *SR_Tx_* = 64 GBd and *SR_Rx_* ∈ {8, 16, 32} GBd. In those cases, there will be no misalignment in time and the obtained symbols will be correctly decoded, thus leading to average Euclidean distances equivalent to that obtained when *SR_Rx_* = *SR_Tx_*. However, when *SR_Rx_* is a multiple of *SR_Tx_*, some of the decoded symbols come from inter-symbol times which will be observed as an increased Euclidean distance. Clearly, in both cases, the number of symbols that will be obtained at the Rx will not match, i.e., some symbols will be lost when *SR_Rx_* < *SR_Tx_* and more symbols will be obtained when *SR_Rx_* > *SR_Tx_*.

**Figure 3:**
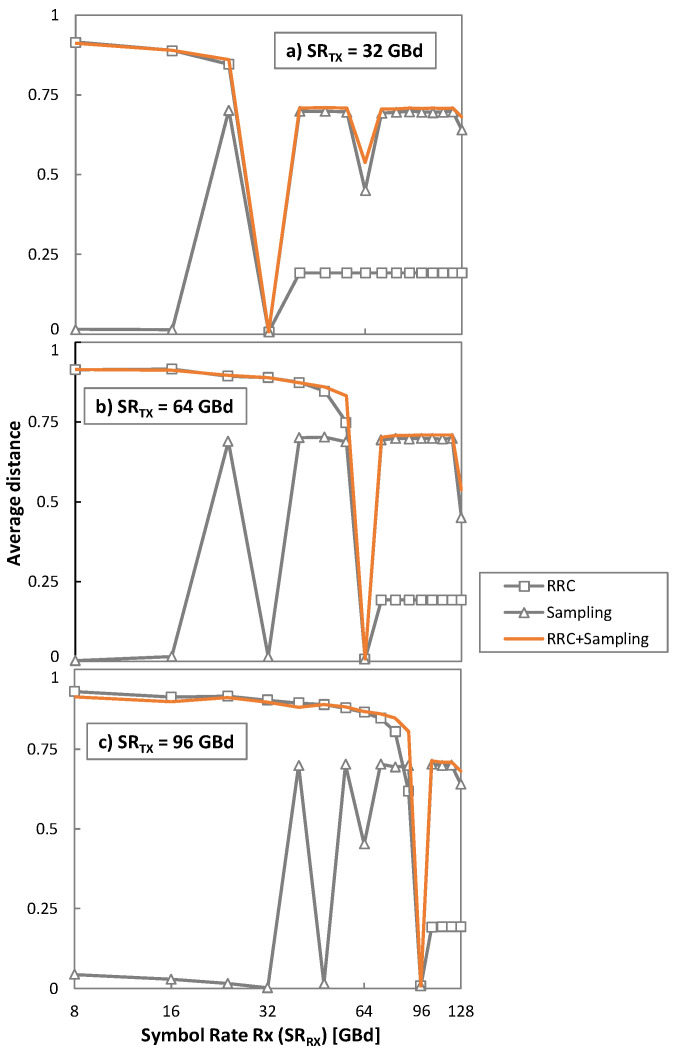
Average Euclidean distance as a function of the *SR_Rx_* for three different *SR_Tx_* considering a B2B scenario.

The plots in Figure 3 show the average Euclidean distance in the analyzed B2B scenario as a function of *SR_RX_*, for 32, 64, 96 GBd. The combined effect and the two individual ones (different RRC filters and the sampling step) are shown separately for a better understanding of the conducted analysis. The following two cases can be separately analyzed: (*i*) *SR_Rx_* < *SR_Tx_* and (*ii*) *SR_Rx_* > *SR_Tx_*. When *SR_Rx_* < *SR_Tx_*, the RRC filter at the receiver is too narrow and ISI appears; consequently, the decoded symbols are far from their original position on the constellation and the Euclidean distance to the closest centroids increases. On the contrary, if *SR_Rx_* > *SR_Tx_*, the RRC filter is wider, so the different sampling effect dominates.

From the results in Figure 3, one can observe that only when *SR_Rx_* = *SR_Tx_* will the average Euclidean distance be minimal. In addition, as the expected centroids are defined for every MF, the average Euclidean distance will be minimized when the right one is selected. Our algorithm for identifying the SR and the MF of a received optical signal can be summarized as follows:

**Hypothesis** **1.** *Given a signal at the Rx side, its configuration, in terms of the SR and MF, can be identified by finding the combination that minimizes the Euclidean distance from the symbols decoded with every supported SR and the centroids defined by the supported MFs, which can be illustrated as follows:*

(5) $$\underset{\left\{\langle SR,MF\rangle \right\}}{\min}{\bar{d}}_{2}\left(S_{SR},C_{MF}\right)$$

**Figure 4:**
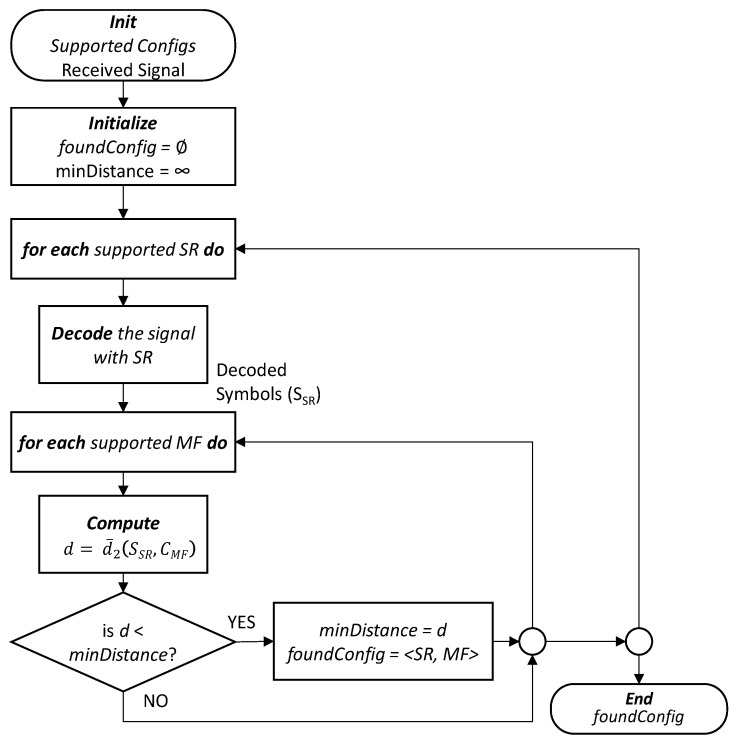
Flowchart of the MF and SR identification algorithm.

The flowchart presented in Figure 4 defines the algorithm that identifies the configuration of the received signal in terms of the SR and MF and the corresponding pseudocode is presented in Algorithm 1. The algorithm exploits the hypothesis presented above by finding the minimum distance of the symbols for all supported configurations (pairs <SR, MF>). The algorithm receives the signal *s* and configurations supported by the transponder with a *configList* of supported SRs and MFs and it returns the most likely SR and MF configuration. After initialization (lines 1–2 in Algorithm 1), the list of possible symbol rates is obtained from the configuration list (line 3). The algorithm iterates over the supported SRs and, for each SR, it decodes the received signal with that SR to obtain the set *S_SR_* of decoded symbols, i.e., the constellation of symbols in the complex space is obtained (lines 4–5). Using this constellation, the MF that minimizes the average Euclidean distance of the decoded symbols, with the centroids *C_MF_* defined by the MF, is selected from the list of possible MFs for the selected SR (line 6). Then, if the average Euclidean distance is smaller than the minimum obtained so far, the configuration is stored and the minimum distance is updated (lines 7–9). Finally, the most likely configuration is returned (line 10).
**Algorithm 1.** Pseudocode of the MF and SR identification algorithm.**INPUT**: *s*, *configList***OUTPUT:***foundConfig*1: 2: 3: 4: 5: 6: 7: 8: 9: 10:*foundConfig* ← <*MF* = ∅, *SR* = ∅> *minDistance* ← ∞ *SRList* = getSRList(*configList*) **for each** *SR* **in** *SRList* **do**   *S_SR_* ← decodeSignal(*s*, *SR*)   <*MF*, *d*> ← findFittestMF(*S_SR_*, getMFList(*configList*, *SR*))   **if** *d* < *minDistance* **then**     *foundConfig* ← <*MF*, *SR*>     *minDistance* ← *d* **return** *foundConfig*

Assuming the optical Tx produces a configuration that is supported by the optical Rx, the identified configuration is correct provided that the hypothesis is also true. Note that although the hypothesis has been demonstrated for B2B scenarios, the symbols will suffer from distortion induced by LI and NLI noise; therefore, the conditions for which the above hypothesis is met should be investigated.

The time complexity of the algorithm is related to the number of configurations <SR, MF> supported by the BVT and to the complexity of Equation (1). Therefore, the complexity can be approximated using the big *O* notation, as follows:

(6) $$O\left(\left|SR\right|\cdot \left|MF\right|\cdot \left|S_{SR}\left|\cdot \right|C_{MF}\right|\right)$$

In the next section, the algorithm presented in Figure 4 is exhaustively evaluated for several scenarios that include LI and NLI noise for high-order MF and high-speed SRs.

## 4. Illustrative Simulation Results

**Figure 5:**
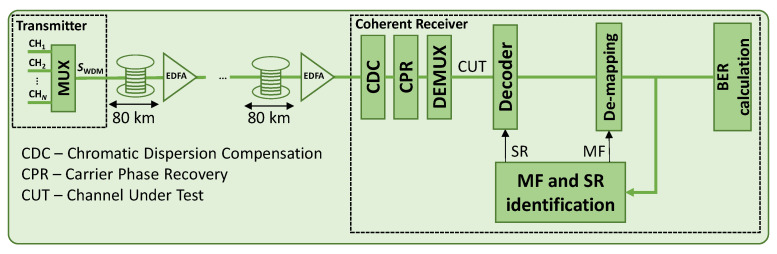
Simulation setup of the WDM system highlighting the DSP part of Rx.

The evaluation of the proposed identification algorithm has been performed through Monte Carlo simulations carried out in MATLAB. We modeled a WDM system with 11 single-carrier channels and considered the central one as the channel under test (CUT), which is impaired by the adjacent channels. A total of 20 equiprobable 2^13^ PRBS were generated, which translate into 1638 and 1365 symbols when 32QAM and 64QAM are used, respectively. Next, the modulated signal is shaped by an RRC filter with a 0.06 roll-off factor. Single polarization 32 and 64QAM signals and SRs of 32, 64, and 96 GBd, which have a low tolerance to NLI noise, were considered. The SSMF and LEAF spans are modeled, as shown in Table 1 [27], and the pulse propagation is modeled by solving the nonlinear Schrödinger equation using the split-step Fourier method (SSFM) with a step size of 100 m. We considered fiber spans of 80 km with an erbium-doped fiber amplifier (EDFA) at the end of each one, which fully recovers the fiber losses; it is characterized by a noise of 4.5 dB. An ideal optical coherent Rx is considered, with ideal chromatic dispersion (CD) compensation and carrier phase recovery. To evaluate the system performance, we measured the bit error rate (BER) by performing direct error counting. The main blocks of the WDM system are depicted in Figure 5, where the relation of the MF and SR identification algorithm with the rest of the blocks is shown.

**Figure 6:**
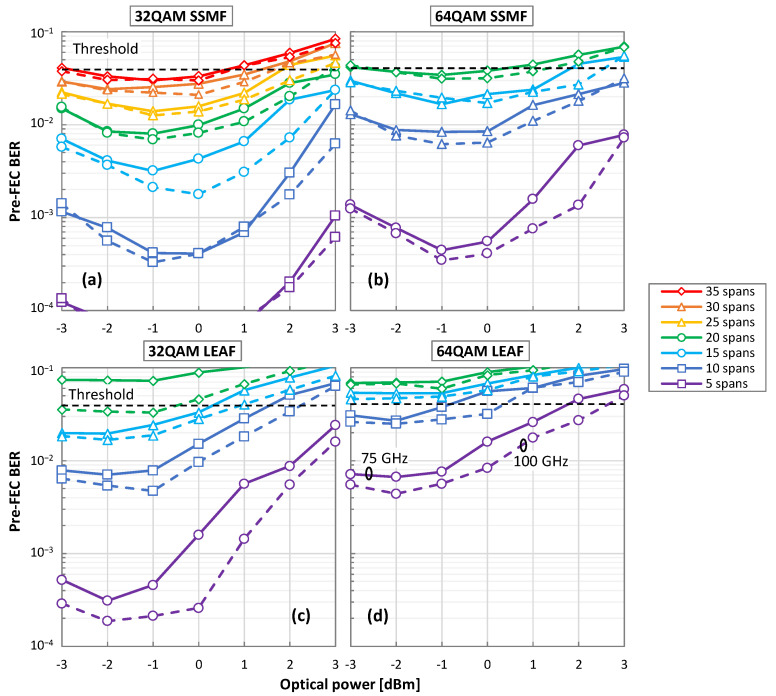
Pre-FEC BER as a function of optical power for 32QAM/SSMF (**a**), 64QAM/SSMF (**b**), 32QAM/LEAF (**c**) and 64AM/LEAF (**d**) over different fiber spans and optical channel spacing.

Before evaluating the proposed MF and SR identification algorithm, we first investigate the maximum reach that each configuration can support and aim to find the suitable range of applicability that will lead to a useful and fair accuracy analysis. Figure 6 presents the obtained pre-FEC BER as a function of the optical power for (a) 32QAM signals and SSMF spans; (b) 32QAM/LEAF; (c) 64QAM/SSMF; and (d) 64QAM/LEAF. An incremental number of spans and 75 GHz (dashed lines) and 100 GHz (solid lines) channel spacings are considered. In view of these results, we can conclude that a higher BER is obtained when LEAF is used, with respect to using an SSMF, due to the smaller CD (*D*_SSMF_ >> *D*_LEAF_) and, consequently, the higher impact of NLI noise. In addition, the overall optimal optical power is around −1 dBm for SSMF and LEAF; therefore, we consider this optical power hereafter.

**Figure 7:**
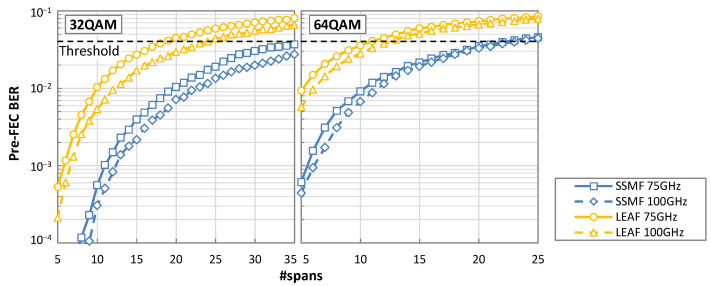
Pre-FEC BER as a function of the number of spans.

Figure 7 shows the pre-FEC BER versus the number of spans for the several cases investigated. Considering a pre-FEC BER threshold of 4 × 10^−2^, we can conclude that for 32QAM and LEAF spans, the signal can reach up to 18 and 24 spans with 75 and 100 GHz channel spacing, respectively. Although, the pre-FEC BER remains below the threshold even after transmission along 35 SSMF spans. In the case of 64QAM, the signal can reach up to 22 and 23 spans of SSMF, and up to 11 and 13 spans of LEAF, when setting the channel spacing to 75 and 100 GHz channels, respectively.

We can now evaluate the accuracy of the proposed algorithm, which entails identifying the scenarios with which the hypothesis stated in Section 3 can be confirmed. The algorithm was applied to identify the signal configuration (i.e., 32QAM or 64QAM and 64GBd for the generated sequences). It should be noted that the algorithm processes a received signal after collecting a large enough number of symbols to produce accurate identification results; it should also be noted that the complexity of the identification algorithm is related to this number. In particular, with the number of symbols generated for 32QAM and 64QAM, and assuming that the BVT supports three different SRs and three different MFs, the number of operations that the identification algorithm will perform (Equation (6)) is in the order of 10^6^, which makes it suitable for real-time implementation.

**Figure 8:**
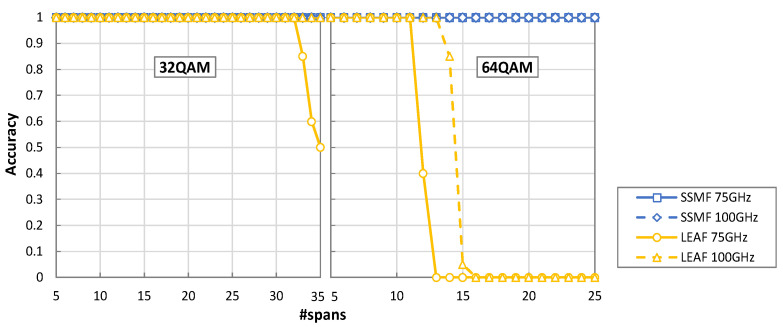
Accuracy as a function of the number of spans.

Figure 8 shows the obtained accuracy of the algorithm as a function of the number of spans for the optical power of −1 dBm. We can observe that the algorithm achieves perfect identification (100% accuracy) for the SSMF spans in all cases investigated. For the LEAF spans and 32QAM signals, the accuracy only decreases from 100% after transmission along 32 spans with a channel spacing of 75 GHz; in the case of 100 GHz channel spacing, perfect performance up to transmission along 35 spans is achieved. When 64QAM signals are considered, the accuracy is 100% until 11 and 13 spans for 75 and 100 GHz, respectively. Note that this is the maximum number of spans imposed by the pre-FEC BER threshold.

**Figure 9:**
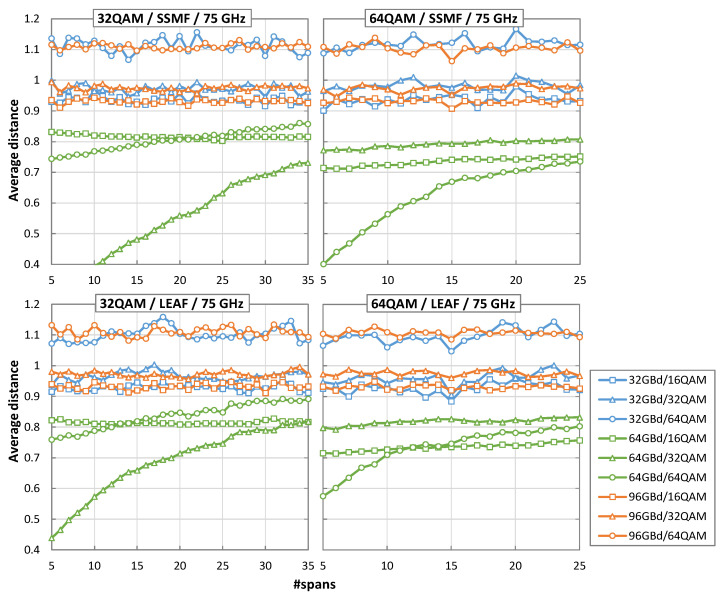
Average distance as a function of number of spans for 32 and 64QAM, SSMF and LEAF, and 75 GHz optical channels.

We now analyze the obtained average Euclidean distances for the different scenarios to identify the conditions where our hypothesis is true, i.e., the minimum average Euclidean distance from the received symbols to the centroids defined by the selected MF is obtained when the signal is decoded with the SR and MF used at the Tx side. Figure 9 plots the average Euclidean distance for 32QAM and 64QAM, and also for the more restrictive 75 GHz channels spacing case, when the received signal is decoded with 32, 64 and 96 GBd and computed for 16, 32, and 64 QAM. We observe that the Euclidean distance for the correct SR/MF is minimal in the entire range of spans until the noise (mainly NLI noise) causes the received signal to exceed the pre-FEC BER threshold. Importantly, the hypothesis is always true when the pre-FEC BER is below the threshold, which empirically demonstrates the validity of our hypothesis for a wide range of application scenarios.

Finally, Table 2 illustrates the number of symbols that need to be analyzed for the highest MF supported under the worst-case scenario. As observed, 1365 and 2048 symbols need to be collected for SSMF and LEAF spans, respectively, for 100% accuracy. Table 3 summarizes the results.

## 5. Conclusions

By analyzing the extensive simulation results presented in this chapter, we can conclude that the proposed algorithm for MF and SR identification is accurate and can run in real-time. The algorithm is based on a hypothesis that relates the decoded symbols with the Euclidean distance to the expected centroids defined by each supported MF, and states that this distance should be minimal for the SR and MF used at the Tx side. The hypothesis is supported by the two main effects that impact the Euclidean distance when an optical signal is decoded at the Rx side with SRs and MFs that are different from those used at the Tx side, i.e., (*i*) narrower RRC filter when *SR_Rx_* < *SR_Tx_*; and (*ii*) sampling step when *SR_Rx_* > *SR_Tx_*.

The simulation results of a coherent WDM system for realistic scenarios were presented, where the optical signal was impaired by both LI and NLI noise. The 32QAM and 64QAM signals, SSMF and LEAF spans, and 75 and 100 GHz channels spacing were considered. The results showed 100% accuracy for the maximum reach imposed by the pre-FEC BER threshold for all the cases investigated.

## Figures and Tables

**Table 1:** Parameters for the SSMF and LEAF spans.

	SSMF	LEAF
Attenuation factor (*α*) [dB/km]	0.21	0.225
Dispersion parameter (*D*) [ps/(nm·km)]	16.8	4.2
Nonlinear parameter (*γ*) [(W·km)^−1^]	1.14	1.3

**Table 2:** Accuracy [%] as a function of number of received symbols for 64AM/75GHz.

#Symbols	Collecting Time @32GBd	SSMF (25 Spans)	LEAF (11 Spans)
512	16 ns	83%	40%
1024	32 ns	99%	75%
1365	42.6 ns	100%	89%
2048	64 ns	100%	100%

**Table 3:** Summary of results.

	Max #Feasible Spans (Pre-FEC BER)			
	32QAM		64QAM	
	75 GHz	100 GHz	75 GHz	100 GHz
SSMF	>35	>35	22	23
LEAF	18	24	11	13
	**Max #Span for 100% Accuracy**			
	**32QAM**		**64QAM**	
	**75 GHz**	**100 GHz**	**75 GHz**	**100 GHz**
SSMF	>35	>35	>25	>25
LEAF	32	>35	11	13
